# Effects of grazing on the relationship between dominant shrubs and understory vegetation along sand dune stability gradient

**DOI:** 10.1371/journal.pone.0308462

**Published:** 2024-11-08

**Authors:** Tiantian Zhu, Wenxu Cao, Xu Li, Lu Hai, Xiulian Zhao, Qinghe Li

**Affiliations:** 1 State Key Laboratory of Efficient Production of Forest Resources, Key Laboratory of Tree Breeding and Cultivation of National Forestry and Grassland Administration, Research Institute of Forestry, Chinese Academy of Forestry, Beijing, China; 2 Experimental Center of Desert Forestry, Chinese Academy of Forestry, Dengkou, China; University of Udine: Universita degli Studi di Udine, ITALY

## Abstract

During the process of dune vegetation restoration, understanding how grazing disturbance affects the relationship between plant species is a critical issue in ecological studies. However, there is insufficient evidence on the changes in the interaction between dominant shrubs and understory vegetation under grazing behavior. We aimed to study how grazing and dune stabilization affects the relationship between *Caragana microphylla* and understory vegetation. We established fencing at various stages of dune stabilization and proceeded to compared the performance indicators (e.g., richness and biomass) and the relative interaction index of understory vegetation and different functional groups along the dune stability under grazing and fencing conditions. Results showed that *C*. *microphylla* had facilitation on understory plants, and increased with dune stability, while the facilitation of *Caragana microphylla* on understory vegetation was stronger under grazing conditions. As sand dune stabilization increases, the facilitation of *C*. *microphylla* on understory vegetation diversity decreases significantly. However, there was no significant difference in the facilitation of *C*. *microphylla* on understory vegetation biomass at different stages of sand dune stabilization. This is related to the survival strategy of perennials being less tolerant to environmental stress than annuals, because grazing increased the richness of both annuals and perennials while reducing the overall biomass, and in the later stages of sand dune stabilization, and the facilitation of *C*. *microphylla* on perennials was higher than on annuals. Our study highlights the importance of the responses of different life-form groups to environmental factors and grazing disturbance during the process of sand dune vegetation restoration, as they play a crucial role in shaping the development of the relationship between understory vegetation and dominant shrubs.

## Introduction

Desertification, a process characterized by the persistent degradation of dryland ecosystems, has been widely acknowledged as a major environmental challenge. It detrimentally impacts the livelihoods and living conditions of populations in numerous countries across the globe, particularly in arid and semi-arid regions. According to the United Nations Convention to Combat Desertification (UNCCD), approximately 30% of the Earth’s land surface is threatened by desertification, affecting over 1.5 billion people worldwide (UNCCD, 2023). This phenomenon is exacerbated by factors such as climate change, unsustainable land management practices, and deforestation, leading to a decrease in land productivity and an increase in vulnerability to extreme weather events.

Planting sand-stabilizing shrubs that have facilitation effects is an effective tool and has been widely used for ecological restoration of control the further expansion of desertification because they use their strong root systems to restrict the flow of sand dunes and promote the restoration of sandy vegetation by reducing wind erosion and water evaporation on the sandy surface and sustaining plant-soil feedback [1–4]. Meanwhile, during the change of dune stabilization stage, the establishment and growth of dominant shrubs have changed the understory vegetation community composition [5]. As a result of these efforts, mobile sand changes from semi-fixed dunes with thin soil crust and low vegetation cover to fixed ones that have high vegetation cover. In the process of dune stabilization, the combined impact of dominant shrubs and environmental pressures dictate whether the relationship between these shrubs and the understory vegetation is one of promotion or competition. Furthermore, changes in the interactions among plants play a role in shaping the composition of the vegetation community and steering the course of vegetation recovery and succession. Therefore, this is a well-suited system to study the interspecific relationship of plants. Although most of the previous studies have explored the improvement of soil properties and microclimate by dominant shrubs or climate and environment stress on the relationships between plants respectively, limited literature to date has reported the effects of a dominant shrub on herbs under its canopies in the continental dune.

Grazing is recognized as one of the most important grassland practice in the world, and it is also the biggest disturbance factor affecting vegetation restoration of degraded grassland ecosystem [6]. The activities of grazing animals, including the deposition of feces, selective foraging, and physical damage to plants, all alter the availability of resources such as light and nutrients in the local environment. This, in turn, impacts the regeneration of existing plants and the establishment of new plant species, leading to changes in both plant growth and community composition. At the same time, grazing also affects the relationship between dominant shrubs and understory vegetation, and finally affects the direction and results of vegetation restoration [7]. The dominant shrubs employing internal defensive mechanisms (such as secreting toxic substances) and external defensive structures (like thorns) to safeguard understory plants against grazing by large herbivores. This mechanism can be viewed as a form of mutual aid among plants, also known as associational avoidances, indirect avoidances, and refuges [8, 9]. Nevertheless, In the presence of dominant shrubs, the results of community restoration were constrained by the trend of interspecific relationships influenced by grazing [10–12]. The palatable and unpalatable nurse shrubs can influence the foraging behavior of herbivores, and alter plant-damage probabilities. Thereby, shrub palatability can greatly affect not only the intensity of plant interaction but also the composition and diversity of the understory plant community [12]. Dominant shrubs protect subcanopy plants by canopy shelter and the tolerance of itself to moderate the influence of herbivores, and livestock grazing can cause physical and chemical changes in the soil through manure and trampling [13, 14], which in turn alters plant community composition through soil feedback [15].

The stress-gradient hypothesis (SGH) proposed that with the environmental stress increased, the interspecific relationship gradually changed from competition to facilitation [16], and it has been validated by numerous studies in various ecosystems [17–21]. Consequently, increased grazing pressure will enhance the dependence of undergrowth plants on dominant shrub species. In addition, Some scholars also put forward the improvement of SGH and pointed out that surpassing a critical threshold of external pressures may weaken the facilitative effects offered by these shrubs, and net effect of shrubs on undergrowth plants depends on the functional strategies of different plants and their niche positions, the changes in the level of environment stress and coexisting plant species functional strategies will result in distinct outcomes of plant–plant interactions [22]. Therefore, it is of great significance to understand the influence of grazing disturbance and change on the relationship between dominant plants and undergrowth vegetation for the management of vegetation restoration in degraded grassland.

Nonetheless, the outcome of interactions between dominant shrubs and understory vegetation are also influenced by the distinct functional strategies adopted by various plant functional groups, which include shrubs, forbs, and graminoids, as well as their life-form categories, whether they are annuals or perennials [23, 24]. The response of different plant individual to grazing is different because of the different tolerance and attraction of different tree species to herbivores. For instance, grazing reduces the cover of tall annual plants, which in turn leads to a compensatory increase in shorter plants [25], and there have been findings showing that under grazing conditions, the abundance of early-flowering species increases [26]. Furthermore, under grazing conditions, a dynamic of facilitation or competition is observed among annual plants, perennial plants, and dominant shrubs. Research [27, 28] has pointed out that, compared to annual plants, perennial plants generally have a higher attraction to herbivores. As can be seen, annual and perennial herbaceous plants do not respond identically to environmental changes due to their differing life cycles and survival strategies. This insight assists us in better comprehending the alterations in the relationship between dominant shrubs and understory vegetation under grazing disturbances, thus contributing to a deeper understanding of the pivotal role played by dominant shrubs in the process of ecological restoration.

Hulun Buir, the well-preserved natural grassland pastoral area, is located in the transition zone between arid and semi-arid areas in northern China. During the last century, desertification of large areas of the Hulun Buir grassland pastureland was caused by the degradation of the natural environment, the opening of open heathland, cattle grazing, excavation, and excessive human activity, resulting in the formation of the Hulun Buir Sandy Land. Since the 1990s, the method of plant sand fixation has been adopted to achieve ecological restoration by the Chinese government. Since the implementation of revegetation programs such as tree-planting, fly-sowing, and prohibition of grazing, the sandy land has been restored to varying degrees, from mobile sand to semi-fixed sand, and finally to fixed sand, but recently, due to the increasing grazing pressure, disturbances of cattle and sheep occurred in the recovery area had affected vegetation restoration.

At the study site, *Caragana microphylla*, is a spiny *leguminous* shrub, has been widely planted locally to fix sandy land. It is a dominant pioneer shrub species and has been documented to ameliorate soil fertility and raise moisture conditions, provide a suitable microenvironment for understory plants [4, 29]. In addition, *C*. *microphylla* belongs to the Fabaceae family, to helping ecological restoration in degraded ecosystems [30–33], not only because of their canopy role, but also their additional input of nitrogen, which is an essential limiting nutrient in terrestrial ecosystems. And the ability of Fabaceae to transfer some of the nitrogen they have immobilized to the non- Fabaceae plants they are associated with facilitates the growth of coexisting species [34]. Thus, the remarkable resilience and adaptability of *C*. *microphylla* play a pivotal role in the context of sand dune vegetation restoration. Our investigation into the effects of grazing disturbances on *C*. *microphylla’s* facilitation of understory vegetation during dune rehabilitation not only contributes to a deeper understanding of the ecological processes involved in vegetation recovery but also furnishes scientific grounds and practical guidance for desert control and ecological restoration projects. It represents a crucial research direction in the pursuit of sustainable development.

In order to explore the effects of grazing on shrub-grass relationships at the community level and the responses of annual and perennial plants to dominant shrubs and shrubs under grazing disturbances, we try to answer the following questions:(a) How the facilitative effects of shrubs on the species diversity (richness and Shannon-Wiener index) and biomass of the understory vegetation change across different stages of dune stabilization. will it be affected by grazing? (b) What is the response of plants with different life histories to dune stability stages and grazing? (c) How grazing influences the relationship between annual plants (richness and biomass) and dominant shrubs, as well as between perennial plants (richness and biomass) and shrubs, as the stability of dunes changes? We set up fencing (not affected by grazing) as the comparison of grazing in different dune stabilization stages, and selected the same number of dominant shrubs *C*. *microphylla* and understory vegetation, and delineated the corresponding number of shrub-free areas to compare the influence of grazing on shrub-grass relationship.

## Materials and methods

### Study site

The experiment was established at a Wan Gong forest farm (118°23′E, 49°12′N; 617m above sea level) in the hinterland of Hulun Buir sandy land, and we have obtained permission from the forest management to collect experimental data. The administrative area is under the Prairie Chenbarhu banner of Hulun Buir City, which is also the typical grassland in Hulun Buir sandy land. The mean annual air temperature is −0.5°C, with an average annual frost-free period of 109 days. The mean annual precipitation is 351.3 mm, which is concentrated in June-August. The average annual evaporation and sunshine time are 1385.8 mm and 1414 h, respectively.

In order to prevent the further deterioration and spread of sandy land in the study area, aerial sowing has been carried out continuously every year since 2005, and *C*. *microphylla* have been planted continuously from 2005 to 2015 to fix sandy land, which promotes the fixation of mobile sandy land and accelerates vegetation succession in this area. At present, the sandy dune in this area has been restored to different degrees, forming a vegetation pattern which consists of semi-fixed sandy dune (vegetation coverage 15~40%), fixed sandy dune (vegetation coverage 40~70%) and fixed dunes with soil crust (vegetation coverage>70%). Semi-fixed sandy dune, vegetation coverage is low, and the plant community is greatly affected by wind erosion and sand burial. In the stage of fixed sandy land, there are occasional flaky quicksand on the surface, and compared to semi-fixed sandy land, the vegetation coverage is higher here, and the degree of community disturbance is less severe. Fixed dunes with soil crusts are covered by crusts, and there are abundant biological soil crusts within the community, which significantly enhances vegetation coverage and effectively prevents wind erosion. The vegetation in the study site is composed of desert shrubs and herbaceous plants, the dominant shrubs are *C*. *microphylla*, *Salix psammophila*, *Hedysarum mongolicum*, *Artemisia ordosica*. The herbaceous species were scattered in distribution, most common species are *Oxytropis hailarensis*, *Agropyron cristatum*, *Artemisia scoparia*, *Corispermum stauntonii*, *Artemisia sieversiana*, *Lappula myosotis*, *Elymus dahuricus*, *Medicago falcata*, *Artemisia frigida*, *Dysphania aristate*, *Setaria viridis*.

### Experimental design and data collection

In the study area, we chose the area covered by *C*. *microphylla*, and all sampling tasks were completed in early September 2021. According to the vegetation coverage, semi-fixed dunes (SF, vegetation coverage 25.2±2.25%), fixed dunes (FD, 48.5±3.57%), and crusted fixed dunes (CD, 76.7±3.06%) were selected. Within each stage, large plot of 50m×50m were set up in the enclosed area (not disturbed by grazing) and the adjacent grazing area (disturbed by seasonal grazing behavior with the intensity of 1~2 cow/hm^2^ according to the method of Marques et al. (2001) [35], both with similar soil conditions. Three replicate shrub plots of 10m×10m are set up in each large plot (the distance between quadrats is greater than 20m), and 5 shrubs of equal size are randomly selected for each shrub plots, that is, a total of 6 shrubs plot (total 30 *C*. *microphylla*) are set up in each stable stage (Table 1). Taking the center of the canopy as the origin, the canopy was divided into four uniform quadrants, and 40cm × 40cm herbaceous quadrats were established under each quadrant, and delineate herbaceous quadrats of the same size and number outside the canopy (at least 1m from the edge of the canopy). We recorded and identified the understory plant species, numbers, and the aboveground biomass was collected and dried at 72°C to constant weight for weighing.

**Table 1:** Variety of selected shrub sizes of the three stages of dune stabilization.

Dune stabilization stage	Length (cm)	Width (cm)	Height (cm)
**SF**	118.5±16.04b	89.70±19.87b	112.13±13.76a
**FD**	161±23.55a	131.17±23.70a	150.53±41.49a
**CD**	122.12±22.12b	98.27±27.93b	149.00±36.30b

Note: SF, semi-fixed; FD, fixed dune; and CD, crusted dune; Mean ± sd, N = 30, different letters indicate significant differences in shrub size in different stages of dune stabilization (P<0.05).

### Data analysis

In each herbaceous quadrats, species richness was calculated total number of species. The species Shannon diversity index (*H*) of the communities were calculated according to Stirling & Wilsey(2001) [36].

$$H=-\sum_{i=1}^{S}\left(P_{i}\ln P_{i}\right)$$ (1)

where *S* is the number of species in the quadrat, *P*_*i*_ is the proportion of the number of plants of the *i* plant in the total number of plants.

We calculated the relative interaction index (*RII)* using the following equation to represent the net effect of each individual shrub on the community under the canopy [37]:

$$RII=\frac{X_{w}-X_{0}}{X_{w}+X_{0}}$$ (2)

where *X*_*w*_ and *X*_*0*_ represent the values of each index inside and outside the shrub (with and without shrubs), respectively. The *RII* of paired samples were calculated understory vegetation richness, aboveground biomass, and Shannon index, while the *RII* of different herbal functional groups was calculated based on richness and aboveground biomass. *RII* has defined limits [–1, 1], and positive values indicate facilitation and negative values indicate competition.

All statistical analyses were carried out using R software (R Core Team 2014) and plotting was completed in Origin 2018. To address our first question, we employed a two-factor analysis of variance (ANOVA), using dune stabilization stages and grazing (or fencing) as factors, to analyze the *RII* (richness, biomass, and Shannon-Wiener index) between understory vegetation and dominant shrubs. Subsequently, a three-factor analysis of variance (ANOVA) was employed, with dune stabilization stages, grazing (or fencing), and life-form groups (annuals and perennials) as factors, to analyze their impacts on the species richness and biomass of understory vegetation, thereby addressing our second question. To address our third question, we proceeded with a three-factor analysis of variance (ANOVA), incorporating the dune stabilization stages, grazing (or fencing), and life-form groups (annuals and perennials) as factors, in order to investigate their impacts on the *RII* (richness and biomass) between understory vegetation of different functional groups and dominant shrubs. Each response variable was tested for normality and homogeneity of variance and passed before ANOVA. Tukey post hoc tests for multiple comparisons on ANOVA model results at the *P* <0.05 level.

## Results

The results of two-way ANOVA showed that the *RII* on richness was significantly influenced by the dune stabilization stage and grazing, the *RII* on biomass was significantly influenced by grazing and the interaction between dune stabilization stage and location, the *RII* on Shannon-Wiener index was only influenced by dune stabilization stage (Table 2).

**Table 2:** Results of two-way ANOVA for the effects of dune stabilization, fencing, and their interaction on *RII* for richness, biomass, and Shannon-Wiener index of the understory species (*P < 0.05, **P <0.01, ***P <0.001).

Factor	df	*RII* on richness		*RII* on biomass		*RII* on Shannon-Wiener index	
		F	*P*	F	*P*	F	*P*
**Dune stabilization stage**	2	6.47	<0.01**	2.90	0.06	5.06	<0.01**
**Grazing**	1	13.35	<0.001***	22.57	<0.001***	2.99	0.10
**Dune × Grazing**	2	0.78	0.46	5.54	<0.05**	0.94	0.40

**Fig 1:**
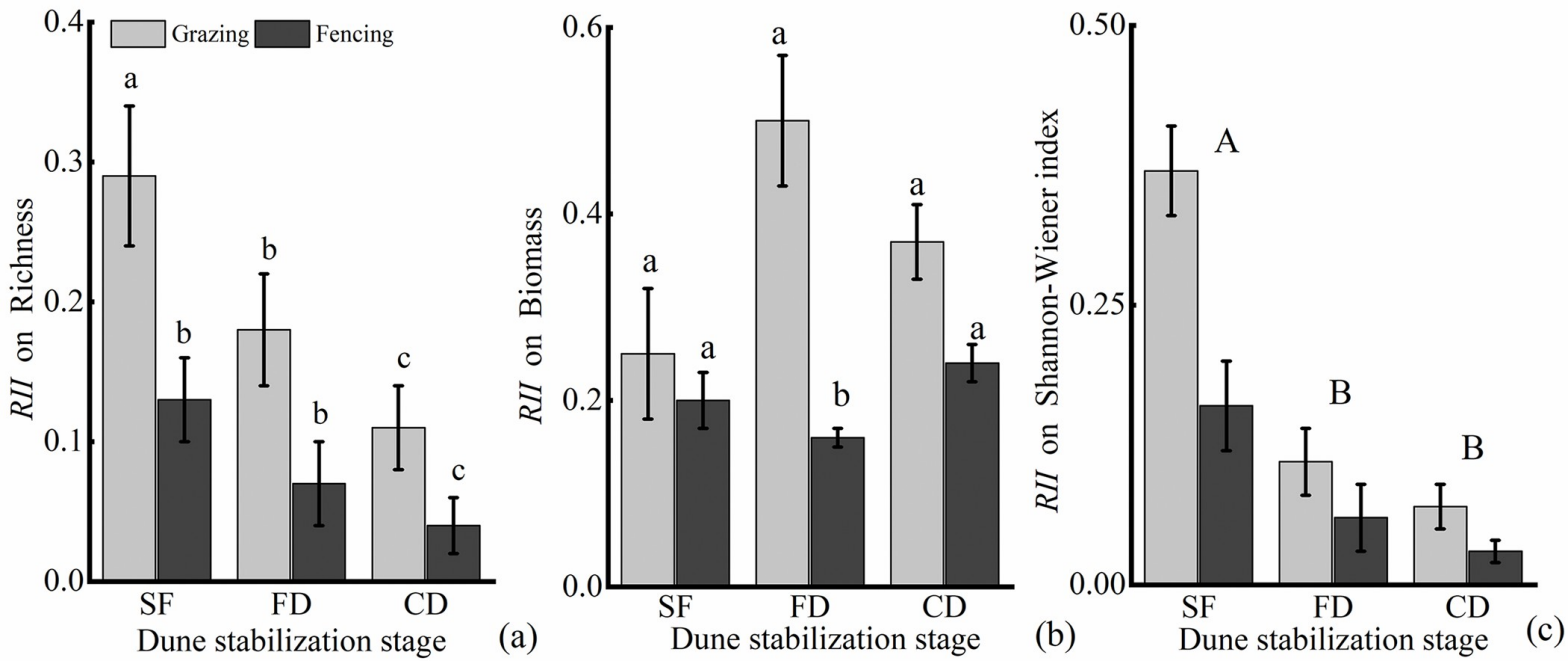
Relative interaction indices for the community-level richness (a), biomass (b), and Shannon-Wiener index (c) of understory species under *C*. *microphylla* under grazing (grey) and fencing (black) treatment across the stages of dune stabilization. SF, semi-fixed dunes; FD, fixed dunes; CD, crusted dunes. The values of *RII* (richness, biomass, and Shannon-Wiener index) represent means±SE (standard error). Those bearing the same letter did not differ significantly (*P*<0.05) using a post hoc Tukey’s test. Different capital letters indicate significant differences between different stages of dune stabilization, while different lowercase letters indicate significant differences between grazing (grazing and fencing) within the same stage of dune stabilization.

The *RII* on richness was decreased with increasing degree of dune stabilization, same trend also was observed on *RII* on Shannon-Wiener index. There is a trend of increased *RII* on biomass, but the difference is not significant in different dune stability stages (Fig 1).

The *RII* on richness and biomass under grazing was higher than fencing. Specifically, the *RII* on richness under grazing was higher that fencing, and the *RII* on biomass was significantly higher than fencing at FD stage, which is increased 210% (Fig 1).

In the open plots (without *C*. *microphylla*), the results of three-way ANOVA shown that the richness and biomass of understory vegetation was significant affects by dune stabilization stage, grazing and different life-form groups, and the biomass was affected by the interaction of three factors (S1 Table in S1 Appendix).

**Fig 2:**
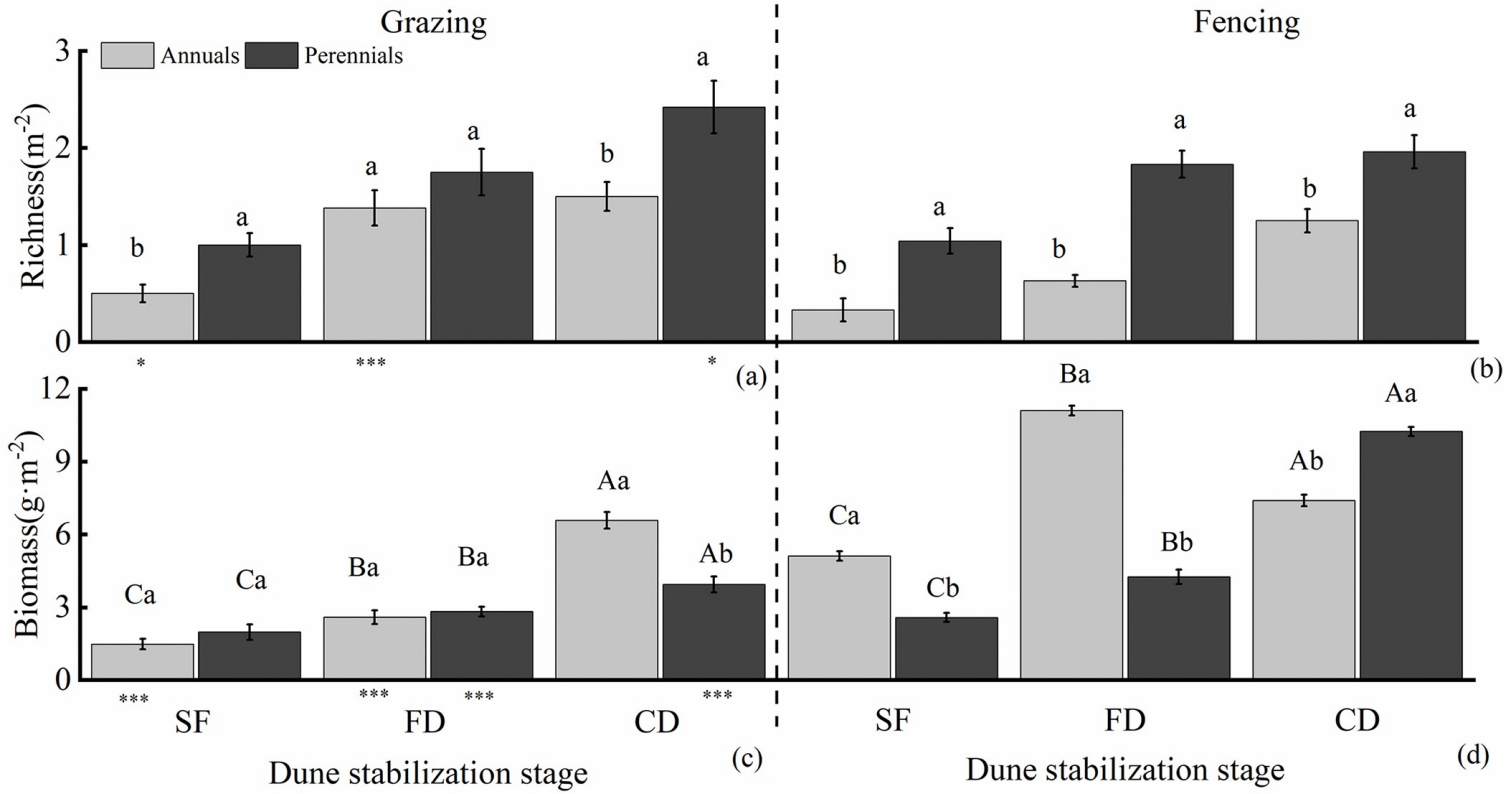
The richness (a and b) and biomass (c and d) of different life-form group under grazing and fencing. SF, semi-fixed dunes; FD, fixed dunes; CD, crusted dunes. The values of richness and biomass represent means±SE (standard error). Those bearing the same letter did not differ significantly (*P*<0.05) using a post hoc Tukey’s test. * indicates significant differences between same life-form(annuals or perennials) plants at grazing (or fencing) within the same stage of dune stabilization (*P < 0.05, **P <0.01, ***P <0.001); Different capital letters indicate significant differences between different stages of dune stabilization within the grazing (or fencing) and same life-form group (annuals or perennials); Different lowercase letters indicate significant differences between different life-form groups (annuals or perennials) within the same stage of dune stabilization and the same treatment (grazing or fencing).

During the FD and CD stages, the richness of vegetation was significantly higher than SF stage, with an average increase of 171%. In comparison to fencing, grazing led to a significant increase in richness, averaging an 86% increment. Furthermore, the richness of perennial plants was notably higher than annual plants, showing an average increase of 122% (Fig 2).

Grazing reduces the biomass both annuals and perennials. Specifically, compared with the fencing, the biomass of annual plants was significantly reduced in the SF, and in the FD, the biomass of both annual and perennial plants was significantly reduced. In the CD, the biomass of perennial plants was significantly reduced. The biomass of annuals and perennials was significantly increased with increasing of dune stabilization. In grazing, CD was increased 220% on average than SF. CD was increased 171% on average than SF under fencing (Fig 2).

The results of three-way ANOVA shown that the *RII* on richness was significantly affected by dune stabilization stage, location and interaction of three factors, and the *RII* on biomass was significantly affected by dune stabilization stage, grazing, life-form groups and interaction of three factors (S2 Table in S1 Appendix).

**Fig 3:**
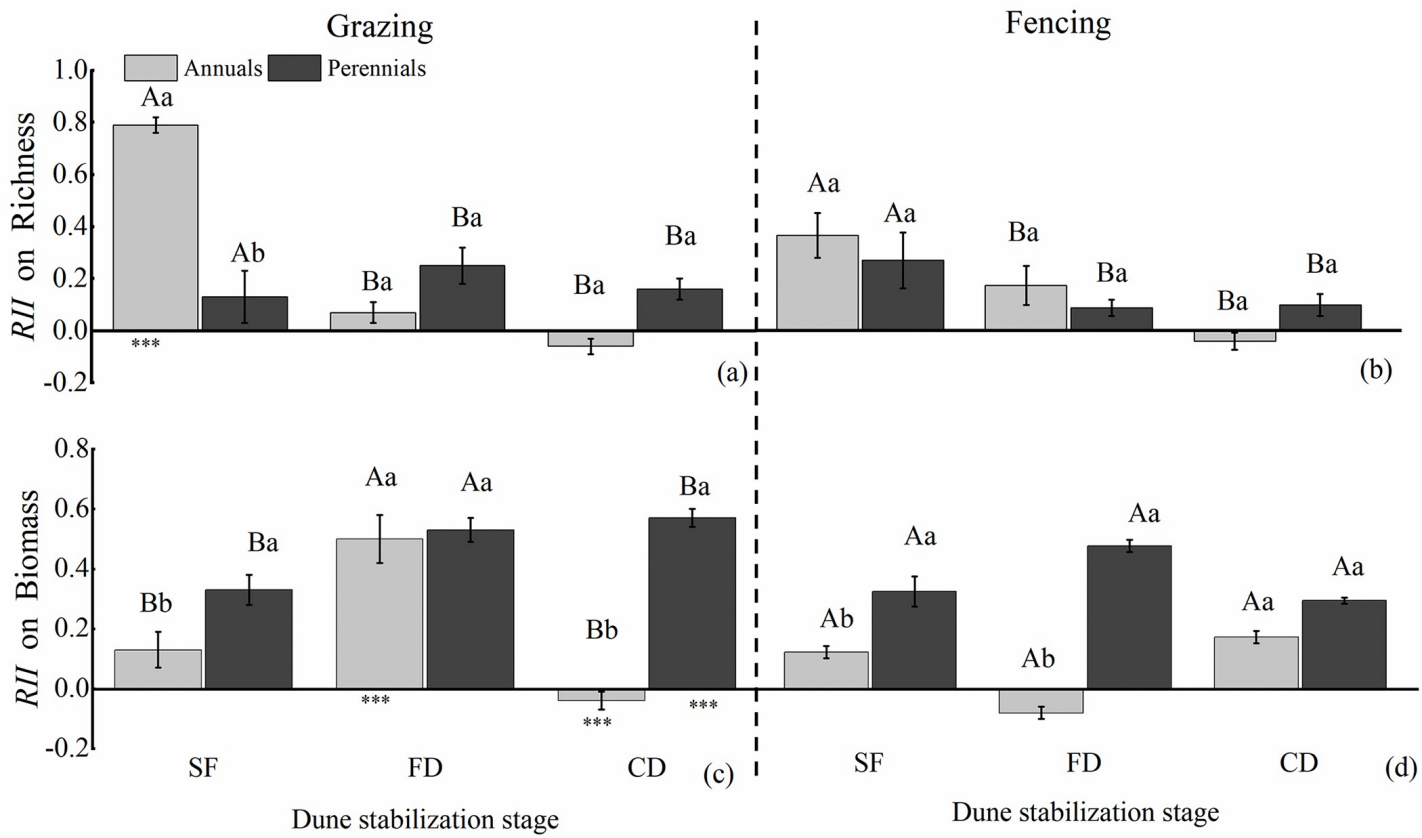
Relative interaction indices (*RII*) on richness (a, b) and biomass (c, d) of the two life-form groups under fencing and grazing. SF, semi-fixed dunes; FD, fixed dunes; CD, crusted dunes. The values of *RII* represent means±SE (standard error). Those bearing the same letter did not differ significantly (*P*<0.05) using a post hoc Tukey’s test. * indicates significant differences between same life-form(annuals or perennials) plants at grazing (or fencing) within the same stage of dune stabilization (*P < 0.05, **P <0.01, ***P <0.001); Different capital letters indicate significant differences between different stages of dune stabilization within the grazing (or fencing) and same life-form group (annuals or perennials); Different lowercase letters indicate significant differences between different life-form groups (annuals or perennials) within the same stage of dune stabilization and the same treatment (grazing or fencing).

There is a significant difference in *RII* on richness between the pre-stabilization (SF) and post-stabilization stages (FD and CD). Compared to fencing, grazing changed the trend of the change in perennial plants with the increasing of dune stability. For grazing, the *RII* on richness of annual plants gradually decreased with the stability of dunes, with the highest value appearing in the SF stage(0.79±0.03), while the *RII* on richness of perennial appeared at its highest value in the FD stage(0.25±0.07), Furthermore, grazing gradually weakens the *RII* on richness of annual plants and enhances the perennials with the enhancement of dune stability (Fig 3).

Under grazing, the *RII* on biomass of perennials was higher than that on annuals, especially in the SF and CD. There were significant differences in *RII* on biomass among different stages under grazing. Specifically, grazing mainly changed the intensity and direction (competition or facilitation) of shrub facilitation on both annuals and perennials in the later stages of dune stabilization (FD and CD). In which, with the increasing of dune stability, there was an increasing trend in *RII* on biomass for both annual and perennial plants under grazing, but the *RII* on biomass of annuals significantly decreased in the post-stabilization stage (CD) (Fig 3).

## Discussion

These results answer our three questions. For first question, under grazing, the facilitation of *C*. *microphylla* on the diversity and biomass of understory herbaceous was higher. Additionally, with the improvement of sand dune environment, the facilitation of *C*. *microphylla* on the diversity of understory herbaceous (richness and Shannon-Wiener index) gradually decreased, but the difference in facilitation on biomass was not significant. For second question, On the one hand, the richness of understory herbaceous (both annuals and perennials) gradually increased with the improvement of sand dune environment. Across all stages of dune stabilization, grazing increases the species richness of understory herbaceous (both annuals and perennials), and the richness of perennials was higher than annuals. On the other hand, grazing reduced the biomass of both annuals and perennials, and with the improvement of sand dune environment, the biomass of both annuals and perennials increased. For third question, from fixed sand dunes to crusted fixed dune, grazing weakened the facilitation intensity of *C*. *microphylla* on the richness of annual plants, while enhancing the facilitation intensity on the richness of perennials. In addition, under grazing, the facilitation of *C*. *microphylla* on the biomass of perennials was higher than annuals, and the influence of *C*. *microphylla* on the biomass of understory vegetation varied significantly among different stages of sand dune stabilization.

Our results indicate that the facilitation of *C*. *microphylla* on the richness of understory vegetation (both annuals and perennials)decreases with the improvement of sand dune environment, which may be related to the different responses of herbaceous with different life-form group to environmental changes, because in areas without shrubs, the richness of understory (both annuals and perennials) gradually increases with the improvement of sand dune stability, and grazing increases annuals and perennials richness. Under grazing, the facilitation of *C*. *microphylla* on understory is more obvious (value was higher), and the richness of perennials is higher than annuals in all stages of sand dune stabilization, indicating that perennials species are more sensitive to changes in sand dune environment and grazing, and are the major contributors to the facilitation effect of *C*. *microphylla*. we also found that under grazing, the facilitation of *C*. *microphylla* on the biomass of perennials was higher than annuals. Furthermore, as the sand dune environment improves, the facilitation of *C*. *microphylla* on perennials gradually increases, while it turns into a competitive relationship (negative value) with annuals. This indicates that under grazing, the facilitation of *C*. *microphylla* on perennials becomes stronger, while the relationship become competitive between *C*. *microphylla* and annuals.

On the one hand, these results are consistent with the stress-gradient hypothesis (SGH), it proposed that the facilitation increases with increasing abiotic/biotic stress in communities, which has been accepted and verified by many experiments [38–41]. The presence of shrubs and grazing behavior significantly influence the composition of herbaceous vegetation communities, Substantial evidence suggests that biotic stress imposed by consumers alters the direction and strength of species interactions between benefactors and beneficiaries in vegetation communities [14, 42–44] Meanwhile, the benefactor–beneficiary interaction tends to shift from negative to positive under low to intermediate stress levels, while gradually diminishing and may eventually disappear under high stress conditions [45]. On the other hand, the enhancement of sand dune stabilization means that the sand dune environment is constantly improving, and the factors of sand burial and water restriction are correspondingly weakening, which will change the vegetation structure and composition [46]. In addition, grazing, as one of the most important disturbances in grassland ecosystems, is also an important factor in changing vegetation diversity and productivity [47]. Environmental stress is the main driving force behind plant interactions. Specifically, environmental pressures can induce dominant shrubs to modify the microenvironment by offering shade or improving soil conditions, thereby promoting the growth and development of understory vegetation. Conversely, these pressures may also lead to competition for resources (such as water and nutrients) by the dominant shrubs, which in turn suppresses the understory plants. Hence, the interplay between species can transition from facilitation to competition (or the reverse) as environmental pressures fluctuate, depending on the adaptive capacities and survival strategies of the differing species [48, 49]. Annuals, characterized as ’r-strategists,’ exhibit rapid growth in response to rainfall events and are less reliant on microenvironmental improvements [50]. compared to nutrient conditions, the germination and growth of annual plants are more influenced by water availability [51]. Thus, annual plants are more tolerant of environmental stress and disturbance. However, due to their poor shade tolerance, they shift from a facilitative to a competitive relationship with dominant shrubs in the later stages of sand dune stabilization [42, 52, 53]. But perennials have weaker resistance to stress and disturbance compared to annuals and they are more susceptible to the effects of environmental changes. Studies have shown that in grassland restoration, grazing and drought conditions can lead to an increase in the abundance of perennial plants compared to annual plants [47].

Regarding biomass, there is no significant effect of different stages of sand dune stabilization on the facilitation of *C*. *microphylla* on the biomass of understory vegetation. However, grazing had a significant effect on this facilitation (S1 Table in S1 Appendix). This means that grazing has a greater impact on the facilitation of *C*. *microphylla* than the changes in sand dune environment, and, due to the significant interaction between the stages of dune stabilization and grazing, the impact of grazing is not uniform across all stages of dune stabilization (S1 Table in S1 Appendix). This implies that the impact of grazing on vegetation varies under different environmental stress conditions. Analyzing in the context of field realities, during the early stages of dune restoration, when sand burial and wind erosion are more severe and the palatability of understory vegetation is poor, compounded by harsh conditions unsuitable for animal activity, the influence of grazing on the biomass of understory vegetation may be relatively minor. Conversely, in the later stages of dune restoration, as environmental stress eases and the diversity and palatability of understory vegetation improve, livestock tend to preferentially select such areas for grazing activities, thereby intensifying the impact of grazing on vegetation biomass. These factors are all potential reasons underlying the significant interaction observed between stages of dune stabilization and grazing.

In addition, the facilitation of *C*. *microphylla* on annuals and perennials biomass showed opposite changes along the sand dune stabilization gradient. (Fig 3). This turnover in facilitation of *C*. *microphylla* of annuals and perennials was also associated with different functional strategy. Annual plants typically grow rapidly, have short life cycles, and excel at swiftly exploiting transient available resources such as water and nutrients. In contrast, perennial plants possess greater shade tolerance and more efficient resource conservation mechanisms, including deeper root systems than annuals, which enable them to tap into groundwater [54]. In the context of the presence of *C*. *microphylla*, these distinct survival strategies imply that annual plants derive greater benefits in the early stages of dune environments where resource competition is particularly fierce. However, as dunes stabilize over time, annuals face increased competition from perennials, which, with the amelioration of the dune environment, are better poised to capitalize on their resource-efficient traits and gain more from the facilitation provided by the *C*. *microphylla*. The association between these distinct functional strategies and the variation in *C*. *microphylla’s* facilitative effects along the gradient of dune stabilization lies in how they dictate the sensitivity and adaptive mechanisms of plants in response to grazing and environmental changes. Consequently, this influences the manifestation of *C*. *microphylla’s* facilitation at different stages and across plant types. For instance, during the early phase with more pronounced resource limitations, annual plants, with their rapid growth strategy, might benefit more directly from facilitation. Conversely, in later stages where resources are more abundant, perennials, with their long-term survival strategies and superior environmental adaptability, are better equipped to exploit the facilitative conditions provided by *C*. *microphylla* to their advantage [55].

Furthermore, grazing reduced the biomass of understory annuals and perennials and increased the richness, with the value of perennial vegetation being higher than that of annuals at various stages of dune vegetation recovery. On the one hand, This is in accordance with Bakker et al., (2003) and Olofsson et al., (2008) who proposed that grazing can improve species diversity by increasing soil N availability, meanwhile reducing plant height and biomass as well [56, 57]. Huston (2014) based on the dynamic equilibrium model predicts that under moderate precipitation, species richness peaks at moderate disturbance levels, while at low precipitation, abundance decreases linearly with increasing grazing intensity [58]. On the other hand, in the process of desertification vegetation restoration, precipitation is a vital factor influencing vegetation dynamics, and the presence of dominant shrubs plays a crucial role in optimizing precipitation utilization [59]. In our study, as dune vegetation progressively recovered, the expanding canopy of these dominant shrubs altered the prevailing precipitation patterns. Specifically, during the later stages of dune stabilization, the understory vegetation experienced a reduction in the amount of precipitation reaching the ground, which consequently led to a decline in annual plant populations and a shift towards an increase in perennial vegetation. In addition, Anvar Sanaei’s research [60] suggests that perennials may affect the growth of annuals due to resource constraints, which may also be an important mechanism that causes perennials to outperform annuals. This phenomenon also reflects the complex interplay between vegetation structure, precipitation distribution, and plant community composition in rehabilitating decertified ecosystems.

There is still a point that cannot be overlooked, we found that current grazing is beneficial to promoting the facilitation of shrubs on understory herbaceous diversity. Nevertheless, as the grazing pressure gradient was not established, future research should further identify the threshold of grazing disturbance to better understand its ecological implications.

## Conclusion

Our results document that the dominant shrub *C*. *microphylla* contributed to understory vegetation diversity and biomass throughout the dune stabilization process. As sand dune stabilization increases, the facilitation of *C*. *microphylla* on understory vegetation diversity decreases significantly, while the facilitation of biomass is not significantly affected. Grazing enhances the promotion effect of dominant shrubs. These changes are related to the effects of sand dune environmental changes and grazing disturbance on different life history plants. Grazing increases the richness of annual and perennial plants and decreases the variation in biomass along the sand dune stabilization gradient. At all stages of sand dune stabilization, the richness and biomass of perennials are higher than those of annuals. In addition, from fixed sand dunes to crusted fixed dune, the facilitation of *C*. *microphylla* on the richness of annuals is weakened under grazing conditions, but is strengthened for perennials. The current findings offer suggestions for the local management of future sand dune restoration highlights the complex effects of grazing disturbance and environmental changes on the restoration of degraded grassland vegetation, providing new evidence for the changes in interspecific relationships of plants under stressful environments. In the future, it is important to further explore the intrinsic mechanisms of biotic and abiotic stress-induced facilitation among plants.

## Supporting information

S1 Appendix The results of the three-way ANOVA analysis in the article, where S1 Table is related to Fig 2 and S2 Table is related to Fig 3. (DOCX)

## References

[1] Gómez-Aparicio L. The role of plant interactions in the restoration of degraded ecosystems: A meta-analysis across life-forms and ecosystems. J Ecol. 2009;97: 1202–1214. doi: 10.1111/j.1365-2745.2009.01573.x

[2] Romero Ovalle PE, Bisigato AJ, Campanella MV. Soil erosion facilitates shrub encroachment in Patagonian herbaceous steppes. L Degrad Dev. 2021;32: 3377–3385. doi: 10.1002/ldr.4016

[3] Li YL, Cui JY, Zhang TH, Okuro T, Drake S. Effectiveness of sand-fixing measures on desert land restoration in Kerqin Sandy Land, northern China. Ecol Eng. 2009;35: 118–127. doi: 10.1016/j.ecoleng.2008.09.013

[4] Zhang C, Wang Y, Jia X, Shao M, An Z. Impacts of shrub introduction on soil properties and implications for dryland revegetation. Sci Total Environ. 2020;742: 140498. doi: 10.1016/j.scitotenv.2020.140498 32623167

[5] Aerts R, Maes W, November E, Behailu M, Poesen J, Deckers J, et al. Surface runoff and seed trapping efficiency of shrubs in a regenerating semiarid woodland in northern Ethiopia. Catena. 2006;65: 61–70. doi: 10.1016/j.catena.2005.09.004

[6] Davies KW, Copeland SM, Bates JD. Grazing effects on shrub-induced resource islands and herbaceous vegetation heterogeneity in sagebrush-steppe communities. Glob Ecol Conserv. 2022;35: e02106. doi: 10.1016/j.gecco.2022.e02106

[7] Kelemen A, Tölgyesi C, Valkó O, Deák B, Miglécz T, Fekete R, et al. Density-dependent plant–plant interactions triggered by grazing. Front Plant Sci. 2019;10: 1–6. doi: 10.3389/fpls.2019.00876 31333709 PMC6624794

[8] Smit C, Vandenberghe C, den Ouden J, Mueller-Schaerer H. Nurse plants, tree saplings and grazing pressure: changes in facilitation along a biotic environmental gradient. Oecologia. 2007;152: 265–273. doi: 10.1007/s00442-006-0650-6 17279351

[9] Milchunas DG, Noy-Meir I. Grazing refuges, external avoidance of herbivory and plant diversity. Oikos. 2002;99: 113–130. doi: 10.1034/j.1600-0706.2002.990112.x

[10] Forey E, Touzard B, Michalet R. Does disturbance drive the collapse of biotic interactions at the severe end of a diversity-biomass gradient? Plant Ecol. 2010;206: 287–295. doi: 10.1007/s11258-009-9642-z

[11] Peláez M, Dirzo R, Fernandes GW, Perea R. Nurse plant size and biotic stress determine quantity and quality of plant facilitation in oak savannas. For Ecol Manage. 2019;437: 435–442. doi: 10.1016/j.foreco.2019.02.010

[12] Baraza E, Zamora R, Hodar JA. Conditional outcomes in plant-herbivore interactions: neighbours matter. OIKOS. 2006;113: 148–156. doi: 10.1111/j.0030-1299.2006.14265.x

[13] Schrama M, Veen GF (Ciska), Bakker ES (Liesbeth), Ruifrok JL, Bakker JP, Olff H. An integrated perspective to explain nitrogen mineralization in grazed ecosystems. Perspect PLANT Ecol Evol Syst. 2013;15: 32–44. doi: 10.1016/j.ppees.2012.12.001

[14] Rahmanian S, Ejtehadi H, Farzam M, Hejda M, Memariani F, Pyšek P. Does the intensive grazing and aridity change the relations between the dominant shrub Artemisia kopetdaghensis and plants under its canopies? Ecol Evol. 2021;11: 14115–14124. doi: 10.1002/ece3.8124 34707844 PMC8525166

[15] Wang Y, Heberling G, Goerzen E, Miehe G, Seeber E, Wesche K. Combined effects of livestock grazing and abiotic environment on vegetation and soils of grasslands across Tibet. Appl Veg Sci. 2017;20: 327–339. doi: 10.1111/avsc.12312

[16] Holmgren M, Scheffer M. Strong facilitation in mild environments: The stress gradient hypothesis revisited. J Ecol. 2010;98: 1269–1275. doi: 10.1111/j.1365-2745.2010.01709.x

[17] Armas C, Rodríguez-Echeverría S, Pugnaire FI. A field test of the stress-gradient hypothesis along an aridity gradient. J Veg Sci. 2011;22: 818–827. doi: 10.1111/j.1654-1103.2011.01301.x

[18] Mcintire EJB, Fajardo A. Facilitation as a ubiquitous driver of biodiversity. New Phytol. 2014. doi: 10.1111/nph.12478 24102266

[19] He Q, Bertness MD, Altieri AH. Global shifts towards positive species interactions with increasing environmental stress. Ecol Lett. 2013;16: 695–706. doi: 10.1111/ele.12080 23363430

[20] Graff P, Aguiar MR. Testing the role of biotic stress in the stress gradient hypothesis. Processes and patterns in arid rangelands. OIKOS. 2011;120: 1023–1030. doi: 10.1111/j.1600-0706.2010.19059.x

[21] Berdugo M, Maestre FT, Kefi S, Gross N, Le Bagousse-Pinguet Y, Soliveres S. Aridity preferences alter the relative importance of abiotic and biotic drivers on plant species abundance in global drylands. J Ecol. 2019;107: 190–202. doi: 10.1111/1365-2745.13006

[22] Grime G.P. Vegetation classification by reference to strategies. Nature. 1974;250: 26–31.

[23] García-Cervigón AI, Linares JC, Aibar P, Olano JM. Facilitation promotes changes in leaf economics traits of a perennial forb. Oecologia. 2015;179: 103–116. doi: 10.1007/s00442-015-3312-8 25903388

[24] Bai Y, Zhang Y, Michalet R, She W, Jia X, Qin S. Responses of different herb life-history groups to a dominant shrub species along a dune stabilization gradient. Basic Appl Ecol. 2019;38: 1–12. doi: 10.1016/j.baae.2019.06.001

[25] Sternberg M, Gutman M, Perevolotsky A, Ungar ED, Kigel J. Vegetation response to grazing management in a Mediterranean herbaceous community: A functional group approach. J Appl Ecol. 2000;37: 224–237. doi: 10.1046/j.1365-2664.2000.00491.x

[26] Hadar L, Noy‐Meir I, Perevolotsky A. The effect of shrub clearing and grazing on the composition of a Mediterranean plant community: functional groups versus species. J Veg Sci. 1999;10: 673–682. doi: 10.2307/3237082

[27] Veblen KE. Season- and herbivore-dependent competition and facilitation in a semiarid savanna. Ecology. 2008;89: 1532–1540. doi: 10.1890/07-0973.1 18589518

[28] Rahmanian S, Hejda M, Ejtehadi H, Farzam M, Memariani F, Pyšek P. Effects of livestock grazing on soil, plant functional diversity, and ecological traits vary between regions with different climates in northeastern Iran. Ecol Evol. 2019;9: 8225–8237. doi: 10.1002/ece3.5396 31380085 PMC6662393

[29] Miao R, Jiang D, Musa A, Zhou Q, Guo M, Wang Y. Effectiveness of shrub planting and grazing exclusion on degraded sandy grassland restoration in Horqin sandy land in Inner Mongolia. Ecol Eng. 2015;74: 164–173. doi: 10.1016/j.ecoleng.2014.10.004

[30] Temperton VM, Mwangi PN, Scherer-Lorenzen M, Schmid B, Buchmann N. Positive interactions between nitrogen-fixing legumes and four different neighbouring species in a biodiversity experiment. Oecologia. 2007;151: 190–205. doi: 10.1007/s00442-006-0576-z 17048010

[31] Hu GZ, Liu HY, Yin Y, Song ZL. The Role of Legumes in Plant Community Succession of Degraded Grasslands in Northern China. L Degrad Dev. 2016;27: 366–372. doi: 10.1002/ldr.2382

[32] De Deyn GB, Cornelissen JHC, Bardgett RD. Plant functional traits and soil carbon sequestration in contrasting biomes. Ecol Lett. 2008;11: 516–531. doi: 10.1111/j.1461-0248.2008.01164.x 18279352

[33] Ouyang S, Tian Y, Liu Q, Zhang L, Sun Y, Xu X, et al. Symbiotic nitrogen fixation and interspecific transfer by Caragana microphylla in a temperate grassland with 15N dilution technique. Appl Soil Ecol. 2016;108: 221–227. doi: 10.1016/j.apsoil.2016.08.011

[34] Zhang HY, Yu Q, Lü XT, Trumbore SE, Yang JJ, Han XG. Impacts of leguminous shrub encroachment on neighboring grasses include transfer of fixed nitrogen. Oecologia. 2016;180: 1213–1222. doi: 10.1007/s00442-015-3538-5 26747268 PMC4819502

[35] Marques FFC, Buckland ST, Goffin D, Dixon CE, Borchers DL, Mayle BA, et al. Estimating deer abundance from line transect surveys of dung: Sika deer in southern Scotland. J Appl Ecol. 2001;38: 349–363. doi: 10.1046/j.1365-2664.2001.00584.x

[36] Stirling G, Wilsey B. Empirical relationships between species richness, evenness, and proportional diversity. Am Nat. 2001;158: 286–299. doi: 10.1086/321317 18707325

[37] Armas C, Ordiales R, Pugnaire FI. Measuring plant interactions: A new comparative index. Ecology. 2004;85: 2682–2686. doi: 10.1890/03-0650

[38] Elizabeth K. Swanson · Roger L. Sheley · Jeremy J. James. Do shrubs improve reproductive chances of neighbors across soil types in drought? 2019. pp. 79–90. 10.1007/s00442-019-04559-x 31768737

[39] Dohn J, Dembélé F, Karembé M, Moustakas A, Amévor KA, Hanan NP. Tree effects on grass growth in savannas: Competition, facilitation and the stress-gradient hypothesis. J Ecol. 2013;101: 202–209. doi: 10.1111/1365-2745.12010

[40] Reisner MD, Doescher PS, Pyke DA. Stress-gradient hypothesis explains susceptibility to Bromus tectorum invasion and community stability in North America’s semi-arid Artemisia tridentata wyomingensis ecosystems. J Veg Sci. 2015;26: 1212–1224. doi: 10.1111/jvs.12327

[41] Hesse E, O’Brien S, Luján AM, Sanders D, Bayer F, van Veen EM, et al. Stress causes interspecific facilitation within a compost community. Ecol Lett. 2021;24: 2169–2177. doi: 10.1111/ele.13847 34259374

[42] Osem Y, Perevolotsky A, Kigel J. Grazing effect on diversity of annual plant communities in a semi-arid rangeland: Interactions with small-scale spatial and temporal variation in primary productivity. J Ecol. 2002;90: 936–946. doi: 10.1046/j.1365-2745.2002.00730.x

[43] Verwijmeren M, Smit C, Bautista S, Wassen MJ, Rietkerk M. Combined Grazing and Drought Stress Alter the Outcome of Nurse: Beneficiary Interactions in a Semi-arid Ecosystem. Ecosystems. 2019;22: 1295–1307. doi: 10.1007/s10021-019-00336-2

[44] Pajunen AM, Oksanen J, Virtanen R. Impact of shrub canopies on understorey vegetation in western Eurasian tundra. J Veg Sci. 2011;22: 837–846. doi: 10.1111/j.1654-1103.2011.01285.x

[45] Grime JP. Benefits of plant diversity to ecosystems: Immediate, filter and founder effects. J Ecol. 1998;86: 902–910. doi: 10.1046/j.1365-2745.1998.00306.x

[46] Teixeira LH, Weisser W, Ganade G. Facilitation and sand burial affect plant survival during restoration of a tropical coastal sand dune degraded by tourist cars. Restor Ecol. 2016;24: 390–397. doi: 10.1111/rec.12327

[47] Schönbach P, Wan H, Gierus M, Bai Y, Müller K, Lin L, et al. Grassland responses to grazing: Effects of grazing intensity and management system in an Inner Mongolian steppe ecosystem. Plant Soil. 2011;340: 103–115. doi: 10.1007/s11104-010-0366-6

[48] Brooker RW, Maestre FT, Callaway RM, Lortie CL, Cavieres LA, Kunstler G, et al. Facilitation in plant communities: The past, the present, and the future. J Ecol. 2008;96: 18–34. doi: 10.1111/j.1365-2745.2007.01295.x

[49] Liancourt P, Callaway RM, Michalet R. Stress tolerance and competitive-response ability determine the outcome of biotic interactions. Ecology. 2005;86: 1611–1618. doi: 10.1890/04-1398

[50] Gomaa NH, Hegazy AK, Latef AAHA. Facilitation effects of haloxylon salicornicum shrubs on associated understory annuals, and a modified “stress-gradient” hypothesis for droughty times. Plants. 2020;9: 1–14. doi: 10.3390/plants9121726 33297465 PMC7762360

[51] Caccianiga M, Luzzaro A, Pierce S, Ceriani RM, Cerabolini B. The functional basis of a primary succession resolved by CSR classification. Oikos. 2006;112: 10–20. doi: 10.1111/j.0030-1299.2006.14107.x

[52] Pfeiffer M, Langan L, Linstädter A, Martens C, Gaillard C, Ruppert JC, et al. Grazing and aridity reduce perennial grass abundance in semi-arid rangelands–Insights from a trait-based dynamic vegetation model. Ecol Modell. 2019;395: 11–22. doi: 10.1016/j.ecolmodel.2018.12.013

[53] Massante JC, Köbel M, Pinho P, Gerhold P, Branquinho C, Nunes A. Phylogenetic structure of understorey annual and perennial plant species reveals opposing responses to aridity in a Mediterranean biodiversity hotspot. Sci Total Environ. 2021;761: 144018. doi: 10.1016/j.scitotenv.2020.144018 33352349

[54] Bai Y, She W, Michalet R, Zheng J, Qin S, Zhang Y. Benefactor facilitation and beneficiary feedback effects drive shrub-dominated community succession in a semi-arid dune ecosystem. Appl Veg Sci. 2018;21: 595–606. doi: 10.1111/avsc.12388

[55] Steinbauer MJ, Beierkuhnlein C, Arfin Khan MAS, Harter DEV, Irl SDH, Jentsch A, et al. How to differentiate facilitation and environmentally driven co-existence. J Veg Sci. 2016;27: 1071–1079. doi: 10.1111/jvs.12441

[56] Bakker C, Blair JM, Knapp AK. Does resource availability, resource heterogeneity or species turnover mediate changes in plant species richness in grazed grasslands? Oecologia. 2003;137: 385–391. doi: 10.1007/s00442-003-1360-y 12955488

[57] Olofsson J, de Mazancourt C, Crawley MJ. Spatial heterogeneity and plant species richness at different spatial scales under rabbit grazing. Oecologia. 2008;156: 825–834. doi: 10.1007/s00442-008-1038-6 18443826

[58] Huston MA. Disturbance, productivity, and species diversity: empiricism vs. logic in ecological theory. Ecology. 2014;95: 2382–2396. doi: 10.1890/13-1397.1

[59] Bai Y, She W, Zhang Y, Qiao Y, Fu J, Qin S. N enrichment, increased precipitation, and the effect of shrubs collectively shape the plant community in a desert ecosystem in northern China. Sci Total Environ. 2020;716: 135379. doi: 10.1016/j.scitotenv.2019.135379 31839302

[60] Sanaei A, Ali A. What is the role of perennial plants in semi-steppe rangelands? Direct and indirect effects of perennial on annual plant species. Ecol Indic. 2019;98: 389–396. doi: 10.1016/j.ecolind.2018.11.012

