# Supplementary material for: Effects of grazing on the relationship between dominant shrubs and understory vegetation along sand dune stability gradient
Source: PLoS One. 2024 Nov 8;19(11):e0308462. doi: 10.1371/journal.pone.0308462 (PMC11548734; doi:10.1371/journal.pone.0308462)
Supplement: S1 Appendix — (DOCX) [file pone.0308462.s001.docx]

**The results of the three-way ANOVA analysis in the article, where Table S1 is related to Fig 2 and Table S2 is related to Fig 3.**

**S1 Table:** Results of three-way ANOVAs on the effects of dune stabilization, Location, life-form groups and their interactions on *RII* on biomass and richness of subordinate plant species in open plots. Significant results are indicated by asterisks. (**P* < 0.05, ***P* < 0.01, *** *P* <0.001)

| **Factor** | **Df** | **Richness** | | **Biomass** | |
| --- | --- | --- | --- | --- | --- |
|  |  | F | P | F | P |
| **Dune** | 2 | 45.01 | 0.00*** | 212.95 | 0.00*** |
| **Grazing** | 1 | 7.29 | 0.01** | 557.59 | 0.00*** |
| **Group** | 1 | 63.23 | 0.00*** | 88.66 | 0.00*** |
| **Dune×Grazing** | 2 | 1.03 | 0.36 | 85.35 | 0.00*** |
| **Dune×Group** | 2 | 0.51 | 0.60 | 40.52 | 0.00*** |
| **Grazing×Group** | 1 | 2.25 | 0.14 | 26.48 | 0.00*** |
| **Dune×Grazing×Group** | 2 | 2.67 | 0.07 | 155.66 | 0.00*** |
| **Error** | 168 |  |  |  |  |

**S2 Table:** Results of three-way ANOVAs on the effects of dune stabilization, Location, life-form groups and their interactions of *RII* on richness and *RII* on biomass of understory plant species. Significant results are indicated by asterisks. (**P* < 0.05, ***P* < 0.01, *** *P* <0.001)

| **Fctor** | **Df** | ***RII*_richness_** | | ***RII*_biomass_** | |
| --- | --- | --- | --- | --- | --- |
|  |  | **F** | **P** | **F** | **P** |
| **Dune** | 2 | 33.019 | 0.00*** | 13.149 | 0.00*** |
| **Grazing** | 1 | 4.823 | 0.03* | 30.074 | 0.00*** |
| **Group** | 1 | 1.866 | 0.1738 | 146.880 | 0.00*** |
| **Dune×Grazing** | 2 | 1.658 | 0.1935 | 20.145 | 0.00*** |
| **Dune×Group** | 2 | 22.248 | 0.00*** | 4.448 | 0.01** |
| **Grazing×Group** | 1 | 1.124 | 0.2905 | 0.229 | 0.6332 |
| **Dune×Grazing×Group** | 2 | 13.087 | 0.00*** | 44.764 | 0.00*** |
| **Error** | 168 |  |  |  |  |
